# Bioactive fatty acids from non‐conventional lipid sources and their potential application in functional food development

**DOI:** 10.1002/fsn3.3521

**Published:** 2023-07-06

**Authors:** Bessem M. Akonjuen, John O. Onuh, Alberta N. A. Aryee

**Affiliations:** ^1^ Department of Human Ecology, Food Science & Biotechnology Program College of Agriculture, Science and Technology, Delaware State University Dover Delaware USA; ^2^ Department of Food and Nutritional Sciences College of Agriculture, Environment and Nutrition Science, Tuskegee University Tuskegee Alabama USA

**Keywords:** extraction, functional food, health benefits, microalgae, nonconventional lipid sources

## Abstract

There is growing evidence that bioactive fatty acids (BFAs), including eicosapentaenoic acid (EPA; 20:5–3), docosahexaenoic acid (DHA; 22:6–3), and conjugated fatty acids offer multiple biological benefits and constitute ingredients in functional food development. Despite their potential, novel and alternative/nonconventional sources with unique bioactive properties to meet growing demand remain largely unexplored, poorly characterized, and their effects are not well understood. We systematically reviewed the literature to identify studies on alternative sources of BFAs, their functions, extraction, and application in the food and nutraceutical industry. Twenty studies delved into alternate sources such as plants, bacteria, and algae. Six studies found EPA and DHA as the dominant FA in algal sources, while ten studies reported several BFAs from plant sources. Five studies assessed the health benefits of docosapentaenoic acid (DPA), arachidonic acid (ARA), EPA, γ‐linolenic acid (GLA), and linoleic acid (LA). Eleven studies compared the quality of oil recovered by green solvents, pressurized liquid, supercritical fluid, and assisted extraction methods. Three studies assessed the effects of assisted extraction methods and reported that these approaches improved oil yield and quality, but the findings may have limited applicability to other lipid sources. The quality of nonconventional lipids largely depends on extraction techniques. Four studies suggested methods like 1D and 2D NMR spectroscopy, LC‐MS/MS; however, their analytical differences make accurate comparison inadequate. Five studies found that the incorporation of algal and seafood biolipids during product development increased EHA and DHA contents.

## Graphical abstract

Growing evidence and interest in bioactive fatty acids offering multiple biological benefits requires exploration of alternative/nonconventional sources with unique bioactive properties which are properly characterized, and their effects are well understood.

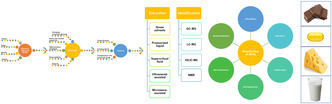

## 1 INTRODUCTION

Bioactive fatty acids (BFAs), such as polyunsaturated fatty acids (PUFAs), conjugated fatty acids, and medium‐chain triglycerides (MCTs), have been extensively studied for their potential health promoting properties in inflammation, cancer, oxidative stress, allergies, diabetes, thrombosis, obesity, hypertension, lipidemia, and other degenerative diseases (Choudhary et al., 2021; Lopes et al., 2020; Lyashenko et al., 2021; Peltomaa et al., 2019). In recent years, there has been a growing interest in BFAs derived from nonconventional lipid sources and their potential applications in various industries, including food, pharmaceuticals, and cosmetics (Akonjuen & Aryee, 2023a, 2023b; Serra et al., 2019). These nonconventional lipid sources include a wide range of plant‐ and microbial‐based oils with unique compositions of BFAs, diverse array of fatty acids (FAs) with distinct nutritional and functional properties (Diomande et al., 2019; Gutiérrez‐Luna et al., 2022). They include seeds and leaves of under‐exploited oleaginous plants (hemp, pomegranate, jacaranda, sea buckthorn, njangsa), algae, and animal sources such as mussel, clam, and krill (Akonjuen & Aryee, 2023b; Alonso‐Esteban et al., 2020; Miller et al., 2020; Otero, 2021; Van Nieuwenhove et al., 2019). These sources are sustainable with possibility of tailored compositions through genetic engineering or cultivation techniques (Aveiro et al., 2020; Prasad et al., 2021).

However, despite the growing interest in BFAs, there is still limited research focusing on their comprehensive study, characteristics, potential health benefits, bioavailability, stability, and applications in various industries. Therefore, in this systematic review, we analyze findings on nonconventional food lipids vis‐à‐vis sources, extraction, characterization, potential health benefits, and applications in the food and nutraceutical industry.

## 2 METHODOLOGY

### 2.1 Literature search

A systematic literature review was performed according to the guidance developed by the European Food Safety Authority (2010), focusing on peer‐reviewed publications in English from 2018 to 2022. Two electronic databases were used to collect the relevant publications: Google Scholar and PubMed. Search strings and selection criteria were predefined and adjusted during the search when necessary.

#### 2.1.1 Search criteria and search strings

Relevant articles published from 2018 to 2022 were selected using the search term “bioactive fatty acids for non‐conventional food lipids” from the Google Scholar and PubMed databases. The words “bioactive fatty acids,” with the exact phrase “bioactive fatty acids,” and at least one of “non‐conventional” “health,” “fats,” “oils,” or “food application” were introduced as filters for an advanced search, and these words could appear anywhere in the article. Meanwhile, words like proteins and carbohydrates were excluded. No filters were used for the authors, publication names, or article types. Reference lists of relevant studies and review articles were searched for additional studies.

#### 2.1.2 Search process

A working database of selected relevant references was created and then screened in the following steps: (a) restricting search to 2018–2022, (b) removing duplicate references, (c) selecting both relevant and “possibly relevant” references, and nonrelevant references excluded based on reading the title, keywords, and abstract, using the selection criteria mentioned earlier, and (d) further evaluating the groups of relevant references and “possibly relevant” references by reading the full texts, and retaining those that met the selection criteria. Furthermore, several valuable articles from the reference lists of relevant studies and review articles were added. All articles meeting the selection criteria were retained and used in this review.

### 2.2 Classification of relevant publications

The studies included in the final selection were classified according to the types of BFAs and health benefits, nonconventional sources, extraction and identification, and application in food and nutraceuticals.

## 3 RESULTS AND DISCUSSION

### 3.1 Literature search

**FIGURE 1:**
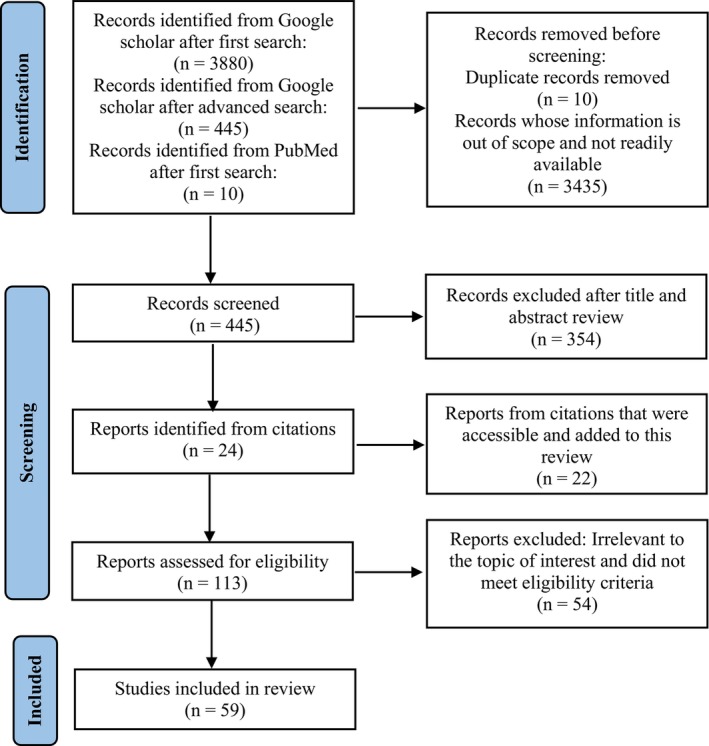
Flow diagram of information search for the systematic review.

The literature search yielded 3880 initial references from Google Scholar and PubMed databases. From the initial set of references, 10 duplicate references were eliminated. A total of 445 abstracts were identified, and 354 were excluded after reviewing titles and abstracts (Figure 1). The remaining articles were screened based on full text, and exclusion was made based on the relevance of the article to the study. Other citations from relevant articles were included.

### 3.2 Overview of studies

**FIGURE 2:**
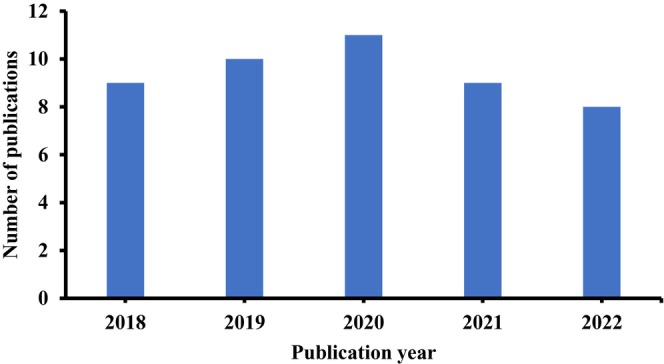
Number of selected publications per year.

An overview of the selected publications is presented in Figure 2 with most articles published in 2020. Nineteen studies focused on nonconventional sources of BFAs, 12 on extraction methods, six on new identification methods, seven on their health benefits, six on application in food, and five on general or other aspects of BFAs.

### 3.3 Bioactive fatty acids from nonconventional lipid sources

BFAs such as linoleic acid and its conjugated form (CLA), alpha‐linolenic acid (ALA, 18:3, n‐3), eicosapentaenoic acid (EPA, 20:5, n‐3), and docosahexaenoic acid (DHA, 22:6, n‐3) have been identified and extensively studied in plant/vegetable, animal, and marine sources (Table 1). FAs such as ARA, EPA, and DHA are converted into eicosanoids which can regulate diverse homeostatic and inflammatory processes linked to numerous diseases including cancer and obesity, maintaining normal heart function and in hypertriglyceridemia management (Miller et al., 2020; Saini & Keum, 2018). Eicosanoids derived from omega‐3 or 6 LC‐PUFAs exhibit anti‐ and pro‐inflammatory activities, respectively (Saini & Keum, 2018). Omega‐3 FAs reduce immune cell production of TNF‐α and other inflammatory cytokines as well as moderate inflammatory gene expression by blocking nuclear factor‐kappa B (NF‐κB), a transcription factor upstream of TNF‐α (Feehan et al., 2022; Little‐Letsinger et al., 2021). However, the emergence of challenges associated with changing consumer demand, niche and market expansion, and reformulation strategies may require diversification and identification of lipids with unique bioactivities for incorporation into functional foods. Nonconventional sources such as novel sources of algae, insects, fruit tree, herbs, and seeds have not been extensively studied and information on evaluation of their bioactivities is often scanty and inadequate.

**TABLE 1:** Sources, extraction of bioactive fatty acids, and their bioactivities.

BFAs	Sources	Extraction method	Bioactivity	References
ALA, LA, oleic acid	Green alga (*Ulva rigida*)	Solvent extraction using dichloromethane and dichloromethane–methanol	Antibacterial effect on *Staphylococcus aureus* ATCC 25923 and *Enterococcus faecalis* ATCC 29212	Ismail et al. (2018)
ALA	Microalga (*Chaetomorpha linum*)	Solvent extraction using chloroform‐methanol	Antibacterial activity against *Vibrio ordalii and Vibrio vulnificus*	Stabili et al. (2019)
EPA	Macroalga (*Cystoseira baccata*)	Accelerated solvent extraction	Antimicrobial activity on *Escherichia coli* and *Staphylococcus aureus*	Otero (2021)
	Red (*P. palmata*) and brown macroalgae (*F. vesiculosus*)	Bligh and Dyer	Potent antioxidant action and inflammatory properties	Lopes et al. (2020)
	Microalga (*Nannochloropsis oceanica*), diatoms and dinoflagellates	Ultrasound‐assisted ethanol, pressurized liquid extraction, microwave assisted, Chloroform: methanol microwave‐assisted ionic liquid extraction	–	Figueiredo et al. (2019); Castejón and Señoráns (2019); Motlagh et al. (2022); Peltomaa et al. (2019)
5,6‐Dihydroxy‐8Z,11Z,14Z,17Z‐eicosatetraenoic acid (5,6‐DiHETE)	Blue back fishes (liver, intestines)	Direct extraction/saponification	Anti‐inflammatory activity by inhibiting hyperpermeability vascular endothelial cells in mice	Kida et al. (2020)
DHA	Fish oil	–	Antiobesity activity by preventing obesity‐induced insulin resistance	Abbott et al. (2020)
	Venus clam (*Marcia opima*) and blood cockles (*Anadara granosa*)	Folch method (chloroform–methanol)	–	Kumar Sethukali and Darshaka Jayasena (2022
EPA, DHA, oleic acid	Seafood (octopus, squid Australian sardine, salmon and school prawns)	Solvent extraction using hexane	Anti‐inflammatory activity	Ahmad et al. (2019)
GLA	“Oyster leaf” and “vegetarian oyster (*Mertensia* spp.) seeds	Direct derivatization of seeds to FAME	–	Lyashenko et al. (2021)
	Hemp seeds	Solvent extraction using n‐hexane‐acetone	–	Alonso‐Esteban et al. (2020)
	Antarctic cyanobacterium *Nostoc* CCC537	Bligh and Dyer method	Antibacterial activity against *Staphylococcus aureus*, *Pseudomonas aeruginosa*, *Salmonella typhi*, *Escherichia coli*, and *Enterobacter aerogenes*	Verma et al. (2021)
SDA	Echium oil, hemp seed oil	Solvent extraction using hexane, acetone, absolute ethanol, ethanol 96%, and n‐hexane–acetone	–	Gutiérrez‐Luna et al. (2022); Prasad et al. (2021) (Alonso‐Esteban et al., 2020)
DHA, SDA	Microalgae (*Emiliania huxleyi*)	Folch method (chloroform–methanol)	Anti‐inflammatory activity by inhibiting 5‐lipoxygenase, the enzyme that catalyzes the formation of inflammatory leukotrienes	Aveiro et al. (2020)
DPA	–	–	Prevention of cardiovascular disease in mice by decreased triglycerides, total cholesterol, non‐HDL cholesterol	Drouin et al. (2019)
LA	Passion fruit, Brazil nuts	Cold pressing using hydraulic presses	Precursor of eicosanoids and regulators of intracellular signaling	Serra et al. (2019)
CLnAs (punicic, jacaric acid)	Pomegranate and jacaranda seeds	Soxhlet extraction using n‐hexane	Anticarcinogenic and anti‐inflammatory activities on cancer cells	Van Nieuwenhove et al. (2019)
Nervonic acid	*Ricinodendron heudelotii* seed oil	Soxhlet extraction using n‐hexane	Biosynthesis of neuronal myelin	Issaka et al. (2019)

#### 3.3.1 Plant sources

Conventional plant sources include food lipids with unique FAs and other components such as sterols, tocols, and carotenoids from flax, chia, soybean, hemp, canola, walnut, and others (Diomande et al., 2019; Van Nieuwenhove et al., 2019). Diomande et al. (2019) reported 26.61% and 53.09% linoleic acid in oil extracted from *Ricinodendron heudelotii* and *Citrullus colocynthis* seeds, respectively. The *R. heudelotii* seed oil contained nervonic acid, a long chain (LC) FA present in the sphingolipids of the white matter of the human brain and is essential for the formation of neuronal myelin. Van Nieuwenhove et al. (2019) reported high content of punicic (omega‐5) and jacaric acids in pomegranate (*Punica granatum*) and jacaranda (*Jacaranda mimosifolia*) seed oils, CLnA, with potent anticarcinogenic components. Rashid (2020) reported 28%–30%, 36%–40%, and 17%–20% omega‐3, 6, and 9 FAs in sea buckthorn berries oil (*Hippophae rhamnoides*), respectively. Prasad et al. (2021) reported that oil from *Echium plantagineum*, Buglossoides, hemp, and black currant seeds were natural sources of stearidonic acid (SDA). SDA is a metabolic intermediate between ALA, EPA, and DHA and has been found to exhibit similar biological functions as EPA and DHA (Aveiro et al., 2020; Prasad et al., 2021). The preventive and therapeutic relevance of SDA relies mainly in its efficient conversion to EPA and DHA in humans, so SDA is considered a “pro‐EPA” (Prasad et al., 2021). Similarly, Gutiérrez‐Luna et al. (2022) reported the presence of SDA (12.09/100 g oil) in echium seed oil. Alonso‐Esteban et al. (2020) reported 2.5%–3.6% γ‐linolenic acid (GLA; 18:3, n‐6) and 15%–20% ALA in hemp seed oil, and Taaifi et al. (2021) found LA in four different cultivars of Morocco‐sourced hemp seed oil. Serra et al. (2019) reported high PUFA levels, especially LA in Amazonian fruits such as passion fruit seed and Brazil nut oils. Lyashenko et al. (2021) assessed various *Mertensia* species, including *M. sibirica* and *M. maritima* spp. Asiatica and observed that the oil extracted from these plants contained 18.7% and 22.8% GLA, respectively. Other studies reported up to 41% α‐eleostearic acid (α‐ESA), a conjugated PUFA in njangsa (Ricinodendron heudelotii) seed oil (Arrey et al., 2022). These could serve as novel sources of BFA‐rich oils, which could be utilized by the pharmaceutical and food or nutraceutical industries in product development and reformulation.

#### 3.3.2 Algal sources

Microalgae are autotrophic microorganisms that absorb CO_2_, light, and inorganic nutrients to produce primary metabolites such as lipids, carbohydrates, proteins, and pigments (Peltomaa et al., 2019). Microalgae can be cultivated under regulated conditions, enabling biomass production with a stable biochemical composition while reducing the possibility of contamination (Marsol‐Vall et al., 2022). Additionally, microalgae can produce bioactive substances such as PUFAs that exhibit health properties (Choudhary et al., 2021; Ramesh Kumar et al., 2019; Saadaoui et al., 2020).

Consumption of seafood products, including macroalgae, has increased worldwide as awareness of their nutritional value and health benefits continue to grow (Lopes et al., 2020; Marsol‐Vall et al., 2022; Rey et al., 2019). Deepali et al. (2021) and Lopes et al. (2020) reported the production of a broad spectrum of BFAs from green (*Ulva rigida* and *Codium tomentosum*), red (*Gracilaria gracilis*, *Palmaria palmata*, and *Porphyra dioica*), and brown (*Fucus vesiculosus*) macroalgae constituting a valuable resource of bioactive lipids with varied potential applications. Oil from marine algae contains EPA and DHA, are equally as effective as fish oil for reducing triglycerides, however, DHA from microalgal sources has been linked to decreases in low‐density lipoprotein cholesterol (LDL‐C) (Lopes et al., 2020; Ramesh Kumar et al., 2019). Dellatorre et al. (2020) reported that brown macroalgal species, particularly in the sporophyll of *U. pinnatifida*, *Ceramium virgatum*, and *Ulva* sp. contained high amounts of PUFAs such as LA, ALA, EPA, SDA, and ARA, with ARA reaching up to 10 mg/g in *U. pinnatifida* sporophyll. Furthermore, Belattmania et al. (2018) reported high amounts of ~48% LA and ARA in *Cystoseira humilis* (Ochrophyta, Phaeophyceae), a brown seaweed, compared to other brown seaweeds studied from the Atlantic coast of Morocco. Stabili et al. (2019) also reported LA, EPA, ARA, and DHA in the seaweed *Chaetomorpha linum* (Chlorophyta, Cladophorales), accounting for 38.46%, 8.83%, 8.14%, and 2.91% of the total FAs, respectively.

He et al. (2019) discovered that microalgae, including *Isochrysis*, predominantly produce n‐3 FAs in glucolipids and phospholipids, with a higher bioavailability than triglycerides. Castejón and Señoráns (2019) and Peltomaa et al. (2019) reported that *Nannochloropsis* sp. could be considered the most prominent strain in commercial DHA and EPA production, with lipid content that ranges between 25% and 45% of dry biomass weight depending on growth conditions. Compared to other microalgae, *Nannochloropsis* has higher lipid content, making it a more promising candidate for use as a lipid source. Peltomaa et al. (2019) reported higher content of EPA in diatoms, while dinoflagellates had higher content of DHA, regardless of their habitat. Deepali et al. (2021) also isolated GLA from an *Antarctic cyanobacterium*, NostocCCC537. GLA is produced using the enzyme, O_2_‐dependent desaturase, making them the only prokaryotes to do that. It was observed that GLA production in the cyanobacterium was also regulated by changes in phosphate or nitrate levels during growth and temperature.

#### 3.3.3 Marine sources

Edible marine bivalves are grown worldwide due to their high n‐3 FAs, particularly eicosapentaenoic acid (EPA, C20:5 n‐3) and DHA (Khawli et al., 2019; Tan et al., 2020). Kumar Sethukali and Darshaka Jayasena (2022) investigated the FA composition of Venus clams (*Marcia opima*) and blood cockles (*Anadara granosa*) harvested from different locations and reported higher amount of DPA (3.72%) in Venus clams and EPA (3.97%) in blood cockles. Green shell mussel oil is the most valuable marine oil (by price) in the world, containing high levels of DHA and EPA, split between the triacylglycerol and polar lipid classes (Miller et al., 2020). Antarctic krill (*Euphausia superba*) oil is characterized by high concentration of EPA and DHA (Colletti et al., 2021) with multiple health benefits.

#### 3.3.4 Health benefits of bioactive fatty acids

Several studies have investigated the effects of BFAs in regulating various physiological processes, inflammation, cell proliferation, metabolic homeostasis, and the prevention of chronic diseases such as cardiovascular diseases (CVD), stroke, and diabetes (Ahmad et al., 2019; Choudhary et al., 2021; Van Nieuwenhove et al., 2019). Dellatorre et al. (2020) and Stabili et al. (2019) reported the potential of DHA and EPA in preventing CVD, schizophrenia, and Alzheimer's disease. Drouin et al. (2019) also demonstrated the beneficial effects of DPA, EPA, and DHA supplementation on cardiometabolic risk indicators, including triglycerides, cholesterol levels, and antioxidant status in healthy rats. Calder et al. (2019) reviewed the effects of ARA on PUFA metabolism and health‐related disorders and found that increased intake of ARA has not been associated with adverse effects on blood lipids, platelet aggregation, immune function, or inflammation or urinary excretion of ARA metabolites.

ARA and DHA play vital roles in the composition of the brain's phospholipid membrane, triggering anti‐inflammatory reactions, blood clotting, and cell signaling (Shanab et al., 2018). Tallima and El Ridi (2018) reported that ARA conferred fluidity, and flexibility to cell membranes in the nervous system, skeletal muscle, and immune system and exhibit protective potential against various conditions, such as *Schistosoma mansoni* and *S. haematobium* infection, and tumor development. Additionally, SDA inhibited 5‐lipoxygenase, the enzyme that catalyzes the formation of inflammatory leukotrienes from ARA, in a dose‐dependent manner compared to the inhibition levels attained by EPA (Aveiro et al., 2020; Prasad et al., 2021). Ahmad et al. (2019) reported that lipid extracts from Australian seafood such as octopus (*Octopus tetricus*), squid (*Sepioteuthis australis*), Australian sardine (*Sardinops sagax*), salmon (*Salmo salar*), and school prawns (*Penaeus plebejus*) inhibited nitric oxide (NO) and tumor necrosis factor‐alpha (TNF‐α) production in lipopolysaccharide (LPS)‐stimulated RAW 264.7 mouse cells. Although NO is a vasodilator, its overproduction during stimulation of macrophages in an inflammatory response can lead to tissue damage through cytokine‐mediated processes. Kida et al. (2020) also reported that cytochrome P450 metabolite of EPA, 5,6‐dihydroxy‐8Z,11Z,14Z,17Z‐eicosatetraenoic acid (5,6‐DiHETE), a novel bioactive lipid exhibited anti‐inflammatory effects by inhibiting the hyperpermeability of vascular endothelial cells in mice. Alonso‐Esteban et al. (2020) reported the positive effects associated with significant intake of GLA on human health by reducing the synthesis and impede the physiological production of dihomo‐γ‐linolenic acid (DGLA) and ARA, leading to a reduction in the formation of critical cell signaling molecules, including prostaglandins, prostanoids, and prostacyclins.

Moreover, omega‐3‐LCPUFA have been investigated for their immunomodulatory effects through the regulation of proinflammatory cytokines (IL‐1β, IL‐6, IL‐8, and TNF‐α) via the production of eicosanoids including prostaglandins, thromboxanes, leukotrienes, and resolvins (Feehan et al., 2022; Shakoor et al., 2021). Feehan et al. (2022) highlighted the potential benefits of increasing n‐3 FAs in bone health and the prevention of osteoporosis‐related fractures. Yang et al. (2023) also assessed the immune and lipid‐lowering effects of fermented tartary buckwheat oil on high‐fat mice and found that the oil gavage reduced total cholesterol, triglycerides, and LDL‐C, increased HDL‐C levels in the liver and plasma, and improved liver damage. Additionally, Little‐Letsinger et al. (2021) demonstrated that mitigation of radiation induced increases in serum TNF‐α was achieved when mice fed with diets high in omega‐3 FAs were exposed to 0.5‐Gy ^56^Fe or 2.0‐Gy gamma radiation.

Furthermore, supplementation with DHA‐enriched fish oil has been associated with improved insulin sensitivity and prevention of obesity‐induced insulin resistance and type 2 diabetes (Abbott et al., 2020). These findings suggest the importance of BFAs in maintaining healthy adipose tissue functioning and metabolic health.

The antibacterial potential of BFAs, particularly those derived from seaweed has also been explored. Ismail et al. (2018) attributed the inhibition of pathogenic bacteria to the alteration in incubation time and concentration of the bacterial FAs precursors oleic, linoleic, and linolenic acids from the green alga *Ulva rigida*. Stabili et al. (2019) also observed that pure ALA from *C. linum* lipid extract inhibited the growth of *Vibrio ordalii* and *V. vulnificus* in an in vitro assay. Deepali et al. (2021) assessed the antibacterial potential of GLA against *Staphylococcus aureus* ATCC25923, *Pseudomonas aeruginosa* ATCC27853, *Salmonella typhi* MTCC3216, *Escherichia coli* ATCC25992, and *Enterobacter aerogenes* MTCC2822. This study showed that GLA was active against both gram (+) and gram (−) bacterial strains in the order: *S. aureus* > *S. typhi* > *E. coli* > *P. aeruginosa* > *E. aerogenes*.

### 3.4 Lipid extraction

Oils and extracts from algal sources, seeds, and others are primarily from dried biomass using solvents, depending on the polarity of the target component (Amaro et al., 2018) (Table 1). Lipid extraction methods have been extensively described and several novel methods including supercritical fluid extraction (SFE), pressurized liquid extraction (PLE), ultrasound‐ and microwave‐assisted extraction have been developed to improve yield, quality, and selectivity compared to solvent extraction (Alonso‐Esteban et al., 2020; Avato & Tava, 2022).

Novel green technologies and solvents are being developed to replace traditional extraction methods due of increasing knowledge of the risks associated with organic solvents in producing food, cosmetics, and pharmaceuticals (Khawli et al., 2019; Marsol‐Vall et al., 2022). These extraction protocols must be safe, efficient, time‐saving, environmentally friendly, and scalable. Conventionally, the chloroform–methanol mixture (Castejón & Señoráns, 2019) and Bligh and Dyer methods (Tommasi et al., 2018) are often used to recover lipids from algae with high efficiency. Castejón and Señoráns (2019) reported higher lipid recovery (15%) from the biomass of *Nannochloropsis gaditana* with the Folch extraction method compared to Soxhlet (1.5%). However, safety concerns with the use of chloroform make this method unsuitable for food applications stimulating studies into less toxic solvents (Figueiredo et al., 2019; Otero, 2021). Green extraction methods have been shown to improve oil quality and stability by retaining bioactives and reducing oxidation.

#### 3.4.1 Green solvent extraction techniques

Solvent extraction is commonly used to recover oil from various sources (Table 1), and many studies have employed nonvolatile, cheaper, and nontoxic organic solvents as excellent choices for safer extraction (Figueiredo et al., 2019; Otero, 2021; Otero et al., 2019). Furthermore, differences in the polarities of lipid compounds require different solvents with similar polarities (Amaro et al., 2018). Alonso‐Esteban et al. (2020) compared n‐hexane and n‐hexane–acetone extractions and found that the latter improved oil yield with a high GLA content in both hemp and hop seeds and did not affect the quality of the oil. The different solvent extraction methods gave similar FA profile. The authors also performed a direct extraction/saponification of hemp seed to fatty acid methyl ester (FAME) and found considerable reduction in the content of GLA, LA, ALA, and SDA, and higher content of saturated and monounsaturated FAs.

A novel class of nonconventional solvents known as natural deep eutectic solvents (NADES) has emerged over the past few years, with the most studied NADES containing choline chloride (ChCl), carboxylic acids, and hydrogen bond donors (urea, citric acid, succinic acid, and glycerol) (Marsol‐Vall et al., 2022). NADES are similar to conventional solvents but cheaper to produce, less toxic, and often biodegradable. Tommasi et al. (2018) found that lipid extraction from a diatom, *Phaeodactylum tricornutum*, with microwave pretreatment and deep eutectic solvents produced with ChCl, and oxalic acid resulted in high amounts of PUFAs, especially EPA comparable to extracts obtained with the Bligh and Dyer method.

Otero (2021) used accelerated solvent extraction (ASE), with ethanol at 100 bar and 120°C for 10 min, to obtain extracts from four algal species, including *Ulva intestinalis*, *Ulva lactuca*, *Cystoseira baccata*, and *Himanthalia elongate*, and investigated their FA profile, antioxidant, and antibacterial activities. The extracts contained carotenoids and phenols, which displayed antioxidant properties ranging from 28 to 64 μg/mL and about 50% inhibition of *E. coli* and *S. aureus*.

#### 3.4.2 Supercritical fluid extraction (SFE)

Supercritical fluid extraction (SFE) ofen uses green solvents which are sustainable and envronmentally friendly. Extraction by this method involves using temperatures and pressures above the critical points of the solvents, which allows these solvents to switch between the gas and liquid phases (Derwenskus et al., 2019). Several studies have investigated using SC‐CO_2_ alone or with cosolvents to extract oil and other bioactive compounds from plants and microalgae. Barbi et al. (2019) investigated the effect of subcritical propane extraction performed under different conditions to assess the effects of temperature and pressure on the yield, composition, and bioactive components in inajá pulp oil and found that subcritical propane provided fast extractions and high yields, reaching up to 28.66 wt.% at 313 K and 6 MPa compared to conventional Soxhlet extraction.

#### 3.4.3 Pressurized liquid extraction

PLE uses green organic solvents (e.g., ethanol) in small amounts and nitrogen (He et al., 2019). Amaro et al. (2018) developed a continuous pressurized solvent extraction system and obtain carotenoids and PUFAs from *Gloeothece* sp., a prokaryotic microalga which was more efficient at extracting the bioactive components than ultrasound‐assisted extraction. Otero et al. (2019) assessed the effect of PLE using four solvents with different polarities (hexane, ethyl acetate, ethanol, and ethanol:water [1:1]) on the nutritional value, FA profile, and phenolic content of lipids from brown alga *Laminaria ochroleuca*. The highest oil yield was obtained using the ethanol–water mixture. Additionally, the study showed that the extraction temperature influenced oil yield, with a 52% lipid recovery at 160°C compared to 37.5% at 80°C, however, lipid profile was not influenced by temperature and its effect on the bioactivities of the FAs was not reported. Oil extracted using ethanol and ethyl acetate had high amounts of unsaturated FAs and the lowest n‐6:n‐3 FA ratio. Similarly, He et al. (2019) reported that using PLE with n‐hexane gave higher lipid extraction efficiency (34.42%) and total FA recovery value (76.45%) compared to Soxhlet extraction with n‐hexane. In addition, lipid extraction efficiency (38.94%) and total FAs recovery (86.48%) using PLE with ethanol was higher than PLE with n‐hexane. Castejón and Señoráns (2019) showed that PLE using different solvents, alone or combined, enabled the simultaneous extraction and fractionation of neutral and polar lipids such as mono‐, di‐, and triacylglycerols, free fatty acids, and glycolipids from the wet biomass of *Nannochloropsis gaditana*. The authors also reported that PLE with n‐hexane at 120°C yielded extracts containing up to 53% EPA, suggesting that ≥120°C did not affect the FA profile.

#### 3.4.4 Assisted extraction methods

Most microalgae have thick cell walls that are diffcult to penetrate during extraction (Amaro et al., 2018), requiring cell disruption, such as exposure to pulsed electric fields, microwave irradiation, and ultrasound (Motlagh et al., 2022) to improve lipid extraction efficiency. Ultrasound‐assisted extraction (UAE) has been used as a green extraction approach due to its ability to improve lipid recovery and kinetics at reduced temperature, solvent, and extraction time compared to conventional methods (Marsol‐Vall et al., 2022). Figueiredo et al. (2019) investigated the potential benefits of using UAE to facilitate lipid extraction from *Nannochloropsis oceanica* biomass. The study also examined the impact of using different solvent mixtures, including chloroform–methanol, dichloromethane–methanol, dichloromethane–ethanol, and ethanol. The results showed that the use of ultrasound‐assisted ethanol extraction significantly increased lipid and EPA recovery compared to the chloroform–methanol mixture (*p* < .05) and the ethanol‐only extraction.

Microwave‐assisted extraction (MAE) has also been demonstrated to be an effective and straightforward method for extracting lipids from microalgae. Motlagh et al. (2022) reported that, the combination of MAE and ionic liquids alongside tetramethyl ammonium chloride ([TMAm][Cl]) proved to be an effective method for extracting lipids from *N. oceanica*. This approach also led to the transesterification of FAs to EPA.

### 3.5 Identification of bioactive fatty acids

The identification and characterization of BFAs and related lipid components, such as eicosanoids in different matrices are of great interest in product development (Mantzourani & Kokotou, 2022). The conventional approach for determining FA composition utilize gas chromatography (GC) coupled with either flame ionization detection (GC‐FID) which requires the conversion of FAs into the suitable methyl esters (FAMEs) or mass spectrometry (GC–MS) (Castejón & Señoráns, 2019; Otero, 2021; Otero et al., 2019). However, other methods such as liquid chromatography‐mass spectrometry (LC–MS) techniques that do not involve derivatization have been developed to quantify FAs (Mantzourani & Kokotou, 2022). Aveiro et al. (2020) characterized the polar lipidome of farmed *Emiliania huxleyi* RCC1250 (strain AC453) using hydrophobic interaction liquid chromatography (HILIC)‐MS, MS/MS, and FA analysis by GC–MS, to scale up its cultivation (photobioreactors) to industrial levels and reported the occurrence of DHA (17.2%) and SDA (11.0%), in *E. huxleyi*'s lipidome. Similarly, Rey et al. (2019) identified 197 molecular species of polar lipids, including glycolipids, phospholipids, and betaine lipids in *Saccharina latissima* using HILIC‐LC–MS. Stabili et al. (2019) characterized lipid extracts from the seaweed *C. linum* using 2D, 1D, and multidimensional NMR spectroscopy, and thin‐layer chromatography and the identified FFAs, SFAs, MUFAs, and PUFAs, which were further confirmed by GC.

Wang et al. (2020) employed an easy solvent‐mediated (SM) covalent adduct chemical ionization system, modified with a triple quadrupole MS, to distinguish polymethylene‐interrupted PUFAs (PMI) from their analogs in direct methyl ester form and identify and characterize the unique omega‐5 FA profile containing PMI‐PUFAs in Ginkgo and five pine nuts species. The prominent Δ5 desaturated PMI‐PUFAs displayed a characteristic fragmentation pattern at C6‐7, resulting in omega‐diagnostic ions and sharing their fragmentation pattern with methylene‐interrupted PUFAs, which yielded alpha‐diagnostic ions. Chemical standards were not required, making this technique suitable for the straightforward analysis of increasingly popular sources rich in omega‐5, such as pine nuts and ginkgo.

### 3.6 Bioactive fatty acids in food and nutraceuticals

Food and nutraceutical products fortified with bioactive substances have become increasingly popular as people become health conscious, and to ameliorate chronic disease conditions (Tan et al., 2020). The high degree of unsaturation of BFAs increases their susceptibility to oxidation and can result in off‐flavors development, deterioration, and reduce consumer acceptability and potential bioactivity (Colombo et al., 2018). The stability and efficiency of BFAs fortified products and susceptibility to oxidation depend on the method used to incorporate the PUFA oils into the product (Pateiro et al., 2021). The most popular techniques for adding PUFA oils to food products include direct bulk addition, emulsification, and encapsulation, with the latter appearing to be the most favorable strategy (Akonjuen & Aryee, 2023a, 2023b; Pérez‐Palacios et al., 2018; Toker et al., 2018). The encapsulation of oils provides protection during processing and storage (exposure to oxygen and light, pH, temperature, time, etc.), and mask the strong odors associated with these types of products (Gulzar et al., 2020).

Due to their high‐consumption frequency and ideal storage conditions, dairy products are excellent candidates for BFAs fortification. The fortification of skimmed milk with shrimp oil nanoliposomes at 10% (v/v) showed 45.41 and 48.86/100 g of EPA and DHA, respectively, which were also bioaccessible and absorbed in the gastrointestinal tract (Gulzar et al., 2020). In addition, oil encapsulated in liposomes allowed for controlled release during digestion. In the cheese‐making process, fish oil microcapsules were added to the cheese matrix, which improved the binding and retention of DHA, and prolonged shelf life (Colletti et al., 2021).

BFAs have also been incorporated into the increasing popular ready‐to‐cook meat products. Pérez‐Palacios et al. (2018) assessed the effect of fortifying chicken nuggets with cod liver oil stabilized with tocopherol (0.40/100 g oil) and reported 5.96% and 25.83% EPA and DHA contents, respectively, in the nuggets. Toker et al. (2018) investigated the effect of different forms and origins of EPA and DHA, such as free‐flowing powder derived from microalgae containing 17% DHA (170 mg/g), algal oil containing at least 35% DHA (350 mg/g), microencapsulated powder based on fish gelatin containing 54 mg EPA and 35 mg DHA, and food lipid triglycerides, containing 250–320 mg EPA + DHA on the production of dark chocolate. High content of EPA/DHA was determined in the dark chocolate samples incorporated with the free‐flowing (198.7 mg/25 g chocolate) and microencapsulated (201.2 mg/25 g chocolate) powders.

Due to the growing market for n‐3 PUFA dietary supplements, supported by mounting evidence, novel sources, and formulations are being sought (Colletti et al., 2021). These novel oil sources are commercialized in the nutraceutical and pharmaceutical markets in different dosage forms, including soft gels, gummies, capsules, and tablets. Georges et al. (2018) assessed the effects of administering krill oil or a placebo to young athletes for 6 weeks during a clinical trial and found that krill oil‐supplemented athletes had higher levels of interleukin‐2 production and natural killer cell cytotoxic activity 3 h postexercise, due to the PUFAs in the oil.

#### 3.6.1 Impact of bioactive fatty acids on oxidative stability and sensory qualities

Shafi et al. (2019) reported that the incorporation of more than 25% melon seed oil in yogurt negative impacted sensory properties and consumer acceptability. Other studies also reported the influence of “free” BFAs on the oxidative stability and sensory attributes of food products (Gulzar & Benjakul, 2020). Strategies such as the addition of natural or synthetic antioxidants, and encapsulation have been used to minimize oxidation. Akonjuen & Aryee (2023b) reported improved oxidative stability of encapsulated njangsa seed oil (NSO) compared to the “free” NSO. Heck et al. (2018) formulated burgers using free chia seed oil (CSO), CSO with rosemary extracts, and CSO microparticles, and found that burgers produced with CSO microparticles enriched with rosemary showed better oxidative stability than the free and encapsulated forms. Gowda et al. (2018) produced ice cream enriched with microencapsulated flaxseed oil powders and reported that the peroxide and thiobarbituric acid values increased up to 30 and 45 days, respectively; and then gradually decreased up to 120 days. To manage the sensory properties of functional foods containing BFAs, some studies have optimized formulations to balance taste, aroma, and texture attributes, using flavor enhancers or masking agents if needed, conducting sensory testing to evaluate consumer acceptance, and providing clear and accurate information about the BFAs to educate consumers and manage their expectations. Srivastava and Mishra (2021) formulated cookies using different concentrations of sunflower and sesame oil powder and reported that cookies fortified with 40% encapsulated oil had a shelf life of 245 days and were more preferred by consumers. Vargas‐Ramella et al. (2020) produced deer pâté by replacing 50% of pork backfat with microencapsulated tigernut, linseed, and chia oils and observed that the fortified pâtés had high redness and yellowness values, and softer texture.

## 4 CONCLUSION AND FUTURE PERSPECTIVES

Global nutritional deficiencies and associated impact on human health has increased demand for functional foods and ingredients including BFAs. The use of nonconventional sources, such as seaweed, seafood, and underutilized sources, provides alternative and sustainable options. Furthermore, the exploration of novel BFA metabolites and their specific mechanisms of action has shed light on their unique health benefits. Novel extraction and characterization technologies have been developed to increase oil recovery, quality, and facilitate accurate identification. The incorporation of BFAs into functional foods impacts oxidative stability, product quality, and sensory properties. Encapsulation has been shown in numerous studies as an efficient approach and its application in food as a suitable strategy to improve handling, stability, delivery, and controlled release, and bioavailability that yields significant consumer acceptance. Further research and development are needed to explore the full potential of BFAs from nonconventional sources in functional foods. Encapsulation technologies can be further optimized to enhance the stability and sensory characteristics of these FAs. Additionally, studies can focus on the development of novel delivery systems and ensure controlled release and bioavailability of BFAs in the human body. Moreover, investigating the interactions of BFAs with other ingredients, such as antioxidants and flavor enhancers, can help optimize formulations and improve overall sensory experience. With continued advancements, BFAs from nonconventional lipid sources have the potential to revolutionize the functional food industry, providing consumers with healthier and more enjoyable food options.

## AUTHOR CONTRIBUTIONS

**Alberta N.A. Aryee:** Conceptualization, Funding acquisition, Project administration, Supervision, Writing ‐ review and editing. **Bessem M. Akonjuen:** Investigation (equal); writing – original draft (equal). **John O. Onuh:** Writing – review and editing (equal).

## FUNDING INFORMATION

This work is supported by the U.S. Department of Agriculture, National Institute of Food and Agriculture (grant no. 2021–67022‐34148).

## CONFLICT OF INTEREST STATEMENT

The authors declare that they have no competing interests.

## Data Availability

Data available within the article.
